# Prospective Evaluation of Cardiovascular, Pulmonary, and Biochemical Changes Following Minimally Invasive Repair of Pectus Excavatum in Children: Study Protocol

**DOI:** 10.3390/children13091273

**Published:** 2026-09-19

**Authors:** Hanna Grabowska, Michele Torre, Tornike Sologashvili, Michał Szostawicki, Kornel Semeran, Małgorzata Kowalska, Adam Hermanowicz

**Affiliations:** 1 Department of Pediatric Surgery and Urology, Medical University of Bialystok, 15-274 Bialystok, Poland; malgorzata.kowalska@umb.edu.pl (M.K.);; 2 Pediatric Thoracic and Airway Surgery Unit, Istituto di Ricovero e Cura a Carattere Scientifico Giannina Gaslini, 16147 Genoa, Italy; micheletorre@gaslini.org; 3 Cardiac Surgery, Faculty of Medicine, Geneva University Hospitals, 1205 Geneva, Switzerland; tornike.sologashvili@hug.ch; 4 Department of Surgery, Clinical Department of Pediatric Surgery and Urology, School of Public Health, University of Warmia and Mazury in Olsztyn CollegiumMedicum, 10-561 Olsztyn, Poland; michal.szostawicki@uwm.edu.pl; 5 Department of Pediatrics, Endocrinology, Diabetology with Cardiology Divisions, Medical University of Bialystok, 15-274 Bialystok, Poland; kornel.semeran@umb.edu.pl

**Keywords:** pectus excavatum, MIRPE, pediatric surgery, echocardiography, myocardial biomarkers, cardiopulmonary function, quality of life

## Abstract

**Highlights:**

**What are the main findings?**

- Biomarkers of myocardial injury and inflammation are assessed before and after the MIRPE procedure in pediatric patients.
- Cardiac, pulmonary, exercise and quality-of-life parameters are evaluated from baseline through postoperative follow-up.

**What is the implication of the main finding?**

- The findings may elucidate the effects of sternal compression and decompression on the pediatric myocardium during minimally invasive correction of pectus excavatum.
- This may contribute to improved perioperative monitoring and long-term follow-up in children undergoing pectus excavatum repair.

**Abstract:**

Background: Pectus excavatum is the most common congenital chest wall deformity and it may impair cardiovascular function, pulmonary mechanics, exercise capacity, and quality of life. Although minimally invasive repair of pectus excavatum (MIRPE) is the current standard surgical treatment, the perioperative dynamics of myocardial injury, inflammation, and their relationship with functional and echocardiographic changes remain incompletely understood, particularly in pediatric patients. Objective: The objective is to prospectively evaluate the perioperative dynamics of cardiac troponin and the change in right ventricular free-wall longitudinal strain as co-primary endpoints, and—as secondary outcomes—additional biochemical markers, echocardiographic parameters, pulmonary function, exercise capacity, and quality of life in children and adolescents undergoing MIRPE. Methods: This is a single-center, prospective, observational cohort study to be conducted at the Department of Pediatric Surgery and Urology, University Children’s Clinical Hospital, Medical University of Bialystok, Poland. Sixty pediatric patients (approximately 14–18 years of age) undergoing primary minimally invasive repair of pectus excavatum will be enrolled. Biochemical markers, including troponins, natriuretic peptides and inflammatory markers will be assessed before surgery and during the first 72 h postoperatively. Echocardiography, pulmonary function tests (spirometry and body plethysmography), the 20 m shuttle run test (Léger), and quality-of-life assessments will be performed preoperatively and during postoperative follow-up. Longitudinal changes will be analyzed using appropriate statistical methods for repeated measurements. Discussion: This study is expected to provide a comprehensive evaluation of the cardiovascular, pulmonary, biochemical, and functional consequences of minimally invasive repair of pectus excavatum in pediatric patients. The findings may improve the understanding of the perioperative myocardial response, cardiac remodeling, and functional recovery following the MIRPE and contribute to optimizing perioperative assessment and long-term follow-up in this patient population.

## 1. Introduction

Pectus excavatum is the most common congenital deformity of the chest wall. It is estimated to occur in approximately 1 in 300–400 live births and is more prevalent in boys [1]. Traditionally, it was regarded primarily as a cosmetic defect; however, it is now recognized as a condition that may adversely affect cardiopulmonary function, exercise capacity, and psychosocial well-being [2,3].

**Figure 1:**
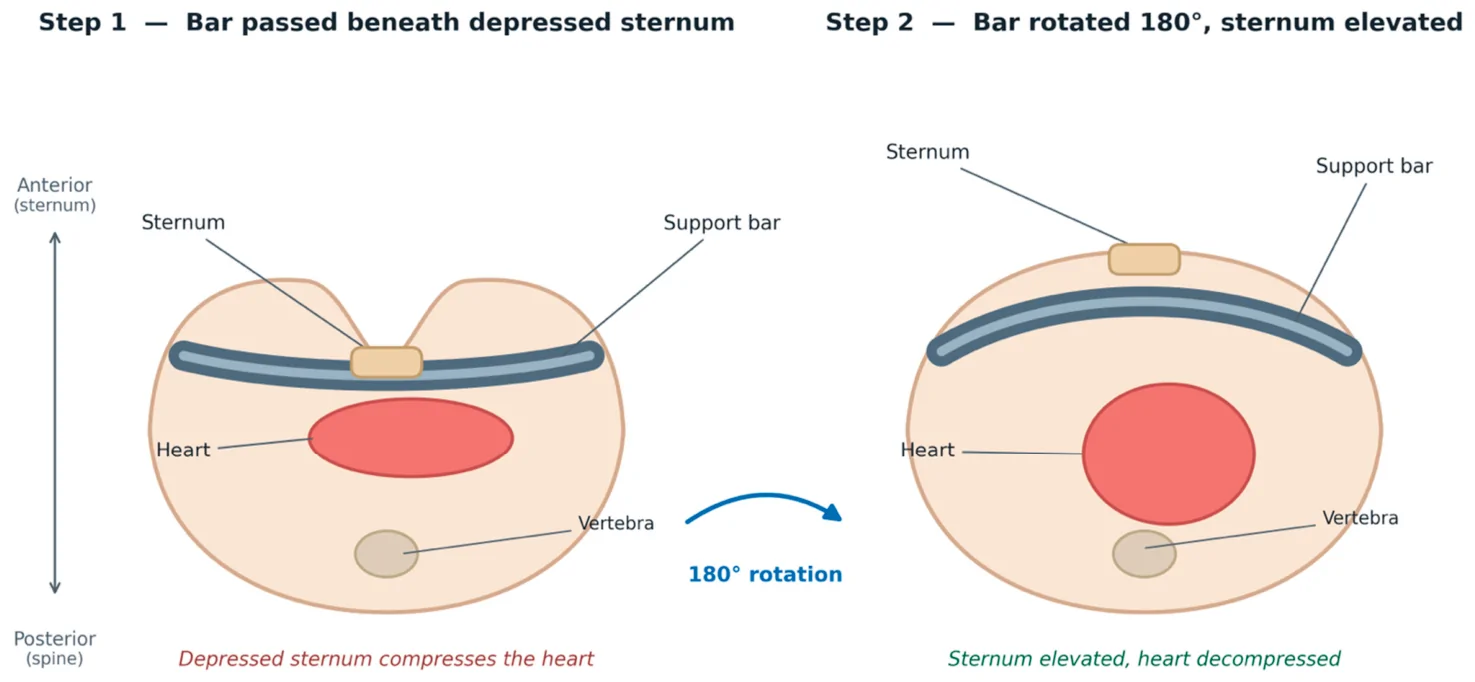
Schematic of minimally invasive repair of pectus excavatum (MIRPE), shown in transverse cross-section. A pre-shaped metal support bar is passed beneath the depressed sternum, which compresses the underlying heart (Step 1). The bar is then rotated 180°, elevating the sternum and anterior chest wall and relieving compression of the heart (Step 2). The bar is left in place for a defined period to maintain the correction and is removed in a subsequent procedure.

The minimally invasive repair of pectus excavatum (MIRPE) has become a widely adopted treatment method due to its favorable cosmetic outcomes and faster recovery compared with the historically used open Ravitch technique. The MIRPE operation involves the substernal placement of one or more contoured metal bars that elevate the depressed sternum and remodel the anterior chest wall [1,4,5]. A schematic representation of this procedure is shown in Figure 1.

Despite its clear advantages, the MIRPE procedure is associated with specific physiological consequences. During substernal bar placement, the pericardium and heart may be subjected to direct compression, while surgical stress and mechanical loading may induce myocardial injury, a systemic inflammatory response, and tissue remodeling, as well as changes in cardiac loading and structure [6,7,8].

The physiological changes occurring during this procedure may be reflected in alterations of biochemical markers of myocardial injury, neurohormonal stress and inflammation. Moreover, the correction of the anterior chest wall may modify cardiac structure, loading conditions, and pulmonary mechanics, potentially improving (or transiently impairing) cardiac function, respiratory function, and exercise capacity [7,8].

The parameters that will be assessed in this study were selected to capture the principal mechanisms expected to occur during and after MIRPE, within a clinically relevant time frame. Troponins were chosen as sensitive and specific markers of myocardial injury, which may result from the mechanical loading and manipulation of the heart during bar placement. Natriuretic peptides (MR-proANP and NT-proBNP) reflect myocardial stress and altered loading conditions associated with the correction of the chest wall. C-reactive protein and interleukin-6 were selected as established markers of the systemic inflammatory response to surgical trauma, which may itself contribute to perioperative myocardial stress. Echocardiography, including right ventricular assessment and strain imaging, was chosen to relate these biochemical changes to structural and functional cardiac adaptation, as the right ventricle is the chamber most affected by sternal compression and its decompression. Finally, pulmonary function, exercise capacity, and health-related quality of life were included to link cardiac findings to clinically meaningful functional and patient-reported outcomes [9].

The existing literature focuses largely on morphological correction, cosmetic outcomes, and global cardiopulmonary improvement after surgery, but comprehensive, prospective pediatric studies that integrally assess all of these domains remain limited [2,3,9]. Furthermore, the perioperative dynamics of myocardial biomarkers and their associations with echocardiographic changes and clinical outcomes have not yet been systematically characterized in children undergoing minimally invasive chest wall correction.

This study aims to provide a comprehensive, prospective evaluation of biochemical, echocardiographic, cardiopulmonary, exercise, and psychosocial parameters in children undergoing the MIRPE procedure at the University Children’s Clinical Hospital in Bialystok, Poland.

### 1.1. Objectives

#### 1.1.1. Hypotheses

- The MIRPE procedure induces a transient perioperative increase in markers of myocardial injury and inflammation, followed by normalization compared with baseline during follow-up.
- Echocardiographic measures of cardiac function, particularly of the right ventricular structure and function and RVFWLS, will improve after a surgical correction of the anterior chest wall.
- Pulmonary function and exercise tolerance will improve after surgery compared with preoperative values.
- Quality of life will significantly improve postoperatively, particularly in the domains of physical functioning and psychosocial well-being.

#### 1.1.2. Primary Objective

The primary objective of this study is to prospectively evaluate the perioperative dynamics of cardiac troponin (a marker of myocardial injury) and the change in right ventricular free-wall longitudinal strain (RVFWLS) (a marker of right ventricular functional adaptation) in pediatric patients undergoing MIRPE. These two co-primary endpoints address the two principal mechanisms of interest: perioperative myocardial injury and the functional response of the right ventricle to relief of sternal compression.

#### 1.1.3. Secondary Objectives

To evaluate the perioperative dynamics of the remaining biochemical markers (NT-proBNP, MR-proANP, CRP, IL-6); additional right ventricular and left ventricular echocardiographic parameters; pulmonary function; exercise capacity; and health-related quality of life, including physical and psychosocial functioning, in pediatric patients undergoing MIRPE.

## 2. Methods

**Figure 2:**
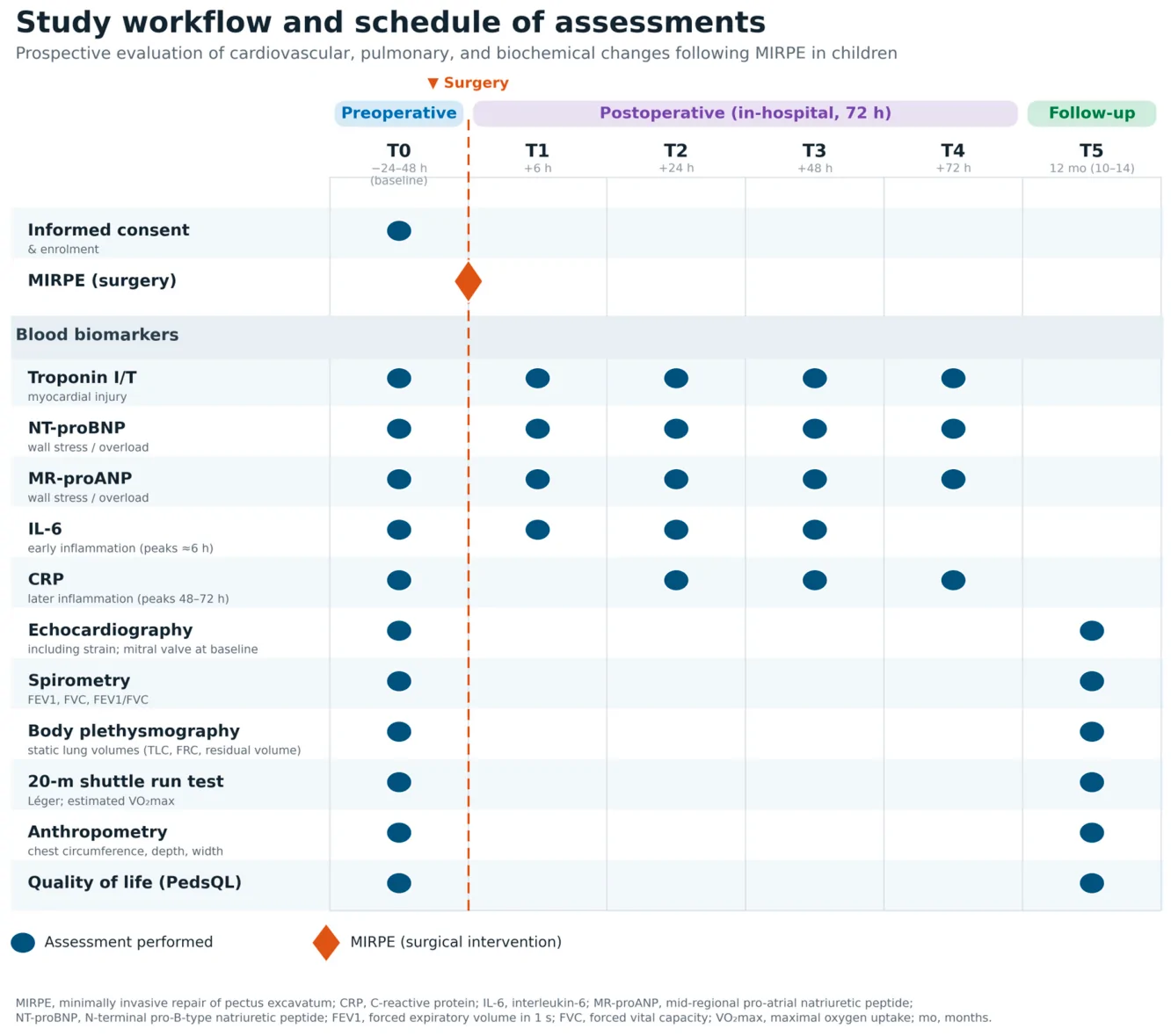
Study workflow and schedule of assessments. Blood biomarkers are sampled according to their kinetics: troponin I/T, NT–proBNP, and MR–proANP at T0–T4; IL–6at T0–T3; and CRP at T0 and T2–T4. Echocardiography, spirometry, body plethysmography (static lung volumes), the 20 m shuttle run test, anthropometry, and quality of life are assessed at baseline and at the 12–month follow–up (T5).

An overview of the study workflow and assessment time points across the perioperative period is shown in Figure 2.

This is a prospective, observational cohort study. All participants will undergo standard-of-care minimally invasive repair of pectus excavatum (MIRPE).

### 2.1. Inclusion Criteria

Age: approximately 14–18 years

1. Diagnosis of pectus excavatum chest wall deformity requiring surgical correction with an implant-based minimally invasive technique.
2. Scheduled for primary MIRPE.
3. Ability of the patient and/or legal guardians to understand the study procedures and provide written informed consent.
4. Willingness and ability to comply with study visits and procedures for the entire follow-up period.

### 2.2. Exclusion Criteria

1. Chest wall deformities other than pectus excavatum (e.g., pectus arcuatum, pectus carinatum).
2. Previous surgical correction of chest wall deformity.
3. Known congenital heart disease or cardiomyopathy with significant hemodynamic impact.
4. Severe chronic pulmonary disease unrelated to chest wall deformity that could confound pulmonary function results.
5. Known systemic inflammatory, autoimmune, or oncologic disease that may affect biomarker levels.
6. Chronic use of medications that significantly influence cardiac or inflammatory biomarkers.
7. Inability to complete echocardiographic or spirometric assessments for technical or cooperation reasons, in the opinion of the investigator.
8. Refusal of consent.

### 2.3. Interventions

1. All patients will undergo standard-of-care MIRPE under general anesthesia. Under thoracoscopic guidance, a substernal tunnel is created, and one or more individually pre-shaped metal bars are passed beneath the sternum and rotated so that the convex side elevates the depressed sternum and remodels the anterior chest wall. In all cases, sternal elevation is additionally supported by the Crane maneuver, in which the sternum is lifted during substernal dissection and bar passage to improve retrosternal visibility and reduce transient direct pressure on the heart. The bar configuration is tailored to the morphology of the deformity, with crossed bars used for C-shaped (“cup-type”) sterna and parallel bars for straighter (“Grand Canyon-type”) sterna. The bars are stabilized to prevent displacement and left in place for a defined period before removal in a subsequent procedure [1,4,5].
2. Perioperative pain control consists of intraoperative cryoanalgesia as the standard approach in most patients, with epidural blockade reserved for a smaller group of patients, typically those with more severe deformities [10]. Further perioperative management, including ventilation strategies, follows institutional standards.
3. The research procedures consist only of additional, scheduled measurements and assessments (blood sampling, echocardiography, spirometry, questionnaires) within clinically acceptable and ethically approved limits.

### 2.4. Outcomes

Time points:

T0: preoperative (within 24–48 h before surgery)

T1: early postoperative (6 h after surgery)

T2: 24 h after surgery

T3: 48 h after surgery

T4: 72 h after surgery

T5: 12 months after surgery (follow-up assessment; permissible window 10–14 months)

Primary outcome measures

The study has two co-primary endpoints:

1. The perioperative change in cardiac troponin (I and/or T) from preoperative baseline across the early postoperative time points (T0–T4).
2. The change in RVFWLS from preoperative baseline to the 12-month follow-up.

All echocardiographic and functional assessments, including the RVFWLS co-primary endpoint, are performed at a single prespecified postoperative follow-up (target 12 months; permissible window 10–14 months) to keep the timing comparable across participants and to capture stable adaptation after chest wall remodeling.

Secondary and exploratory outcome measures

Biochemical markers (change from baseline across postoperative time points):

1. Neurohormonal stress/cardiac overload: MR-proANP and NT-proBNP
2. Inflammatory response: CRP and IL-6

Additional echocardiographic parameters:

1. Right ventricular structure and function: RV dimensions and RV/LV dimension ratio, TAPSE, RV fractional area change (FAC), S′ at the tricuspid annulus (TDI), RV myocardial performance index (MPI, Tei index)
2. Left ventricular systolic and diastolic function, hemodynamic estimates, valvular assessment, LV global longitudinal strain, and additional observations

Pulmonary function and exercise tolerance:

1. Spirometry: FEV1, FVC, FEV1/FVC ratio, and forced expiratory flows
2. Body plethysmography: TLC, FRC, residual volume, and residual volume/TLC ratio, to assess static lung volumes and detect a restrictive or hyperinflation pattern not identifiable by spirometry alone
3. 20 m shuttle run test (Léger test): last completed stage and estimated VO_2_max, with oxygen saturation and heart rate response; perceived exertion (Borg scale) before and after the test

Anthropometric chest parameters (descriptive):

1. Chest circumference at the axillary level (standing position, end of quiet expiration)
2. Depth and width of the chest at maximal depression
3. Haller index and correction index, if CT is available as part of standard care (not mandated by the protocol)

Health-related quality of life:

1. PedsQL questionnaire

### 2.5. Sample Size

The sample size was estimated for the two co-primary endpoints—the perioperative change in cardiac troponin and the change in RVFWLS—using G*Power 3.1, with a two-sided α of 0.05 and a target power (1 − β) of 0.90. In the absence of directly comparable pediatric data for the MIRPE setting, conservative, moderate effect sizes were assumed for both endpoints.

In a prospective pediatric study of the perioperative course of the Nuss procedure, Estefanía et al. enrolled 46 children and demonstrated statistically significant postoperative changes in circulating biomarkers, indicating that clinically relevant perioperative biochemical dynamics are detectable in a cohort of this size [11]. This enrolment is comparable to that of the only pre/post- echocardiographic study in a pediatric population, and a sample of similar magnitude was therefore considered adequate for the troponin co-primary endpoint in the present study.

For the second co-primary endpoint, the change in RVFWLS from baseline to the 12-month follow-up will be analyzed with a paired test (paired *t* test or Wilcoxon signed-rank test). A clinically relevant within-patient change was defined as a standardized effect size of dz = 0.5, that is, a mean change equal to half of the standard deviation of the within-patient differences; under this assumption, 44 patients are required—a value similar to that in the preceding paragraph. To date, no published pediatric study has evaluated RVFWLS by transthoracic echocardiography before and after MIRPE with extended follow-up; the planned study is therefore designed to fill this gap. The only pre/post- echocardiographic study identified in a pediatric population—Raggio IM, Toselli L et al. (Int J Cardiovasc Imaging 2024; n = 43, mean age 15.7 ± 4.0 years)—differs substantially from the present design: it was retrospective, it employed rest and exercise echocardiography focused on diastolic filling parameters rather than RVFWLS, and the postoperative assessment was performed only after bar removal, more than two years following repair [12].

The most demanding co-primary analysis therefore requires 44 patients. Allowing for approximately 25% attrition or incomplete data over the follow-up period, a target enrolment of 60 patients was selected, providing adequate power for both co-primary endpoints. This sample also supports the secondary analyses of the remaining biochemical, echocardiographic, pulmonary, and exercise parameters, which are interpreted as secondary and exploratory. Analyses of associations between biomarkers and functional or echocardiographic parameters (Pearson or Spearman correlation) are considered exploratory and hypothesis-generating; detecting a moderate correlation (r = 0.30) with 0.90 power would require 112 patients, so with the planned sample only moderate-to-strong correlations (|r| ≥ approximately 0.40) can be reliably detected.

### 2.6. Recruitment

Potentially eligible patients will be identified from surgical waiting lists. Information sheets will be provided to families, and informed consent obtained during preoperative visits.

### 2.7. Data Collection, Management, and Analysis

Biochemical markers:

Blood samples will be collected via venipuncture into appropriate tubes according to biomarker requirements. Samples will be processed and stored in accordance with standardized operating procedures, and analyses will be performed using validated immunoassays and standard clinical chemistry analyzers in certified laboratories.

Echocardiography:

Echocardiographic examinations will be performed by experienced pediatric cardiologists, with machines equipped with Doppler, tissue Doppler imaging, and strain imaging capabilities. Key parameters will be measured and averaged over at least three cardiac cycles (or five in the presence of arrhythmias). Strain analysis will be performed using dedicated software. Mitral valve morphology and function, including the presence of mitral valve prolapse and the degree of mitral regurgitation, will be documented at baseline in all participants and considered in the analysis and interpretation of the echocardiographic outcomes.

Pulmonary function and exercise tests:

Pulmonary function will be assessed by spirometry and body plethysmography, performed in all participants at baseline and during follow-up. Both tests will be carried out on a calibrated plethysmograph with daily volume and flow calibration, by trained pulmonary function technicians, in accordance with the ATS/ERS standards for spirometry and for the measurement of lung volumes [13,14]. Measurements will be obtained with the patient seated, wearing a nose clip and breathing through a mouthpiece. For spirometry, at least three acceptable and repeatable forced expiratory maneuvers will be recorded, and the highest valid FEV1 and FVC will be retained. Static lung volumes (TLC, FRC, residual volume) will be measured by body plethysmography and averaged over at least three technically acceptable panting maneuvers. Results will be expressed as absolute values and as z-scores/percent predicted using the Global Lung Function Initiative (GLI) reference equations. A restrictive pattern will be defined as a reduced TLC below the lower limit of normal [15].

Exercise capacity will be assessed using the 20 m shuttle run test (Léger test), conducted according to the standardized protocol, with maximal aerobic capacity (VO_2_max) estimated from the last completed stage and age. Heart rate and pulse oximetry will be monitored, and perceived exertion will be assessed using the Borg scale before and after the test [16].

Anthropometry:

Chest circumference will be measured with a tape at the axillary level (standing position, end of quiet expiration), together with chest width and depth at the level of maximal depression. If available, CT/MRI-derived indices (Haller index, correction index) will be extracted from routine imaging.

Analgesia:

The analgesic method used for each patient (intraoperative cryoanalgesia or epidural blockade) will be recorded as part of data collection and considered in the analysis.

Quality of life and functional status:

Quality of life and functional status will be assessed using validated Polish versions of the PedsQL [17]. Questionnaires will be administered and checked for completeness by trained research staff.

## 3. Statistical Methods

Statistical analyses will be performed using IBM SPSS Statistics (version 32.0). A two-sided *p* value < 0.05 will be considered statistically significant.

The two co-primary endpoints (cardiac troponin and RVFWLS) represent two independent, prespecified primary hypotheses that will be tested and interpreted separately, rather than multiple tests of a single hypothesis. Each addresses a distinct physiological question—myocardial injury and right ventricular functional adaptation—and each will be evaluated at a two-sided α of 0.05, without adjustment for multiplicity across the two endpoints. Accordingly, study success is not defined as joint statistical significance of both endpoints; each hypothesis is assessed on its own merits. Secondary and exploratory analyses will be interpreted as hypothesis-generating.

Continuous variables will be summarized as mean ± standard deviation when normally distributed, or as median with interquartile range when the distribution is skewed. Categorical variables will be reported as absolute frequencies and percentages. The normality of continuous variables will be assessed using the Shapiro–Wilk test together with visual inspection of histograms and Q–Q plots.

Comparisons of preoperative and postoperative values for echocardiographic parameters, pulmonary function, exercise capacity (20 m shuttle run test), exploratory chest anthropometry, and quality-of-life scores will be performed using the paired *t* test or the Wilcoxon signed-rank test, depending on data distribution.

Associations between biochemical markers and functional or echocardiographic parameters will be assessed using the Pearson correlation coefficient for normally distributed variables or the Spearman rank correlation coefficient otherwise. The presence of mitral valve pathology and the analgesic method used (cryoanalgesia or epidural blockade) will be recorded and considered in the analysis and interpretation of the echocardiographic and biochemical outcomes, including sensitivity analyses where appropriate.

### 3.1. Data Management

Study data will be entered directly into a password-protected electronic spreadsheet (Microsoft Excel) with restricted access, stored on secure institutional servers. To minimize data entry errors, range and consistency checks will be applied, and all entered data will be verified against source documents.

Missing data will be reported, and analyses will be based on available data without imputation.

Data monitoring

Given the observational, non-interventional nature of the study and the low additional risk (beyond standard care), a formal independent data monitoring committee (DMC) may not be required. Oversight will be provided by the PI and by the institutional review boards (IRBs).

### 3.2. Harms

All adverse events related to study procedures will be recorded and reported according to institutional policies. Surgical and anesthetic complications, although part of standard care, will also be documented for clinical correlation and safety description.

### 3.3. Ethics and Dissemination

The study received a positive opinion from the Bioethics Committee of the Medical University of Bialystok.

### 3.4. Informed Consent

Written informed consent will be obtained from legal guardians and written assent from children, as appropriate to age and local regulations. Information sheets will clearly describe the study purpose, procedures, potential risks and benefits, data confidentiality, and the voluntary nature of participation, emphasizing that refusal or withdrawal from the study will not affect the standard of care.

### 3.5. Confidentiality

Participant confidentiality will be maintained using coded identifiers. Data will be stored on secure servers with restricted access. Results will be reported in aggregate form.

### 3.6. Dissemination Policy

The results of the study will be published in peer-reviewed international journals, presented at national and international conferences, and used to inform clinical practice guidelines and institutional protocols regarding perioperative assessment and monitoring of children undergoing chest wall deformity correction.

### 3.7. Limitations

Several limitations of this study should be acknowledged.

First, although the sample size was formally estimated for the two co-primary endpoints, the study may remain underpowered for the exploratory correlation analyses between biomarkers and functional or echocardiographic parameters, which would require a larger cohort; these are therefore interpreted as hypothesis-generating.

Second, perioperative analgesia is not uniform across participants, as most patients receive intraoperative cryoanalgesia while a smaller group receives epidural blockade, typically those with more severe deformities. Because the analgesic method may influence inflammatory and stress-related biomarkers and is related to deformity severity, it will be recorded and considered in the analysis.

Finally, missing data will be reported, and analyses will be based on available data without imputation.

### 3.8. Planned Scientific and Practical Impact

The study aims to provide original, comprehensive data on the impact of minimally invasive implant-based correction of chest wall deformities on cardiovascular and respiratory function in children. It will elucidate perioperative and medium-term dynamics of biomarkers of myocardial injury and inflammation to improve the understanding of surgical stress in pediatric patients. Furthermore, it will offer comprehensive echocardiographic characterization (including strain imaging) before and after correction to enhance the knowledge of cardiac adaptation to anatomical remodeling and clarify the relationship between anatomical correction, pulmonary function, exercise tolerance, and patient-reported outcomes. Finally, the study will address the currently limited evidence on the impact of direct cardiac compression and decompression during the MIRPE procedure in children.

## Data Availability

The datasets presented in this article are not readily available because the data are part of an ongoing study. Requests to access the datasets should be directed to the corresponding author.
